# Computerized adaptive testing for the patient evaluation measure (PEM) in patients undergoing cubital tunnel syndrome surgery

**DOI:** 10.1177/17531934231164959

**Published:** 2023-04-17

**Authors:** Joris S. Teunissen, Steven E. R. Hovius, Dietmar J. O. Ulrich, Fadi Issa, Jeremy N. Rodrigues, Conrad J. Harrison

**Affiliations:** 1 Department of Plastic, Reconstructive and Hand Surgery, Radboud University Medical Centre, Radboud Institute for Health Sciences, Nijmegen, Gelderland, The Netherlands; 2 Nufffield Department for Surgical Sciences, University of Oxford, John Radcliffe Hospital, Oxford, UK; 3 Department of Plastic Surgery, Stoke Mandeville Hospital, Buckinghamshire Healthcare NHS Trust, Aylesbury, UK; 4 Clinical Trials Unit, University of Warwick, Coventry, UK; 5 Nuffield Department of Orthopaedics, Rheumatology and Musculoskeletal Sciences, University of Oxford, Oxford, UK

**Keywords:** Patient-Reported Outcome Measures, cubital tunnel syndrome, computerized adaptive testing, surgical procedures

## Abstract

In outcome measures, item response theory (IRT) validation can deliver interval-scaled high-quality measurement that can be harnessed using computerized adaptive tests (CATs) to pose fewer questions to patients. We aimed to develop a CAT by developing an IRT model for the Patient Evaluation Measure (PEM) for patients undergoing cubital tunnel syndrome (CuTS) surgery. Nine hundred and seventy-nine completed PEM responses of patients with CuTS in the United Kingdom Hand Registry were used to develop and calibrate the CAT. Its performance was then evaluated in a simulated cohort of 1000 patients. The CAT reduced the original PEM length from ten to a median of two questions (range two to four), while preserving a high level of precision (median standard error of measurement of 0.27). The mean error between the CAT score and full-length score was 0.08%. A Bland–Altman analysis showed good agreement with no signs of bias. The CAT version of the PEM can substantially reduce patient burden while enhancing construct validity by harnessing IRT for patients undergoing CuTS surgery.

## Introduction

Cubital tunnel syndrome (CuTS) is the second most common compression neuropathy of the upper extremity, with an estimated mean annual incidence of 44/100,000 persons in the United Kingdom (UK) per year (Latinovic et al., 2006). The management of CuTS varies, and conclusive evidence to support the comparative effectiveness of one treatment strategy over another remains lacking (Burahee et al., 2021). High-quality outcome measurement that is ideally low in burden would support future research in CuTS and routine clinical practice outcome measurement.

Patient-reported outcome measures (PROMs) are central to outcome measurement in hand surgery (Wouters et al., 2021), but are not always validated to the highest standards (Wormald et al., 2019). Item response theory (IRT) validation could potentially improve the validation process of PROMs but has rarely been undertaken. Instead of summing the scores of items together, IRT uses probabilistic modelling to map response patterns onto a continuous ‘latent trait’ score (representing the level of hand function, and also known as theta, θ). These θ scores are more accurate, precise and valid than the sum score for the following reasons.

1. They account for individual measurement error by evaluating consistency in a patient's responses; and
2. They are on a continuous scale with equidistant graduations instead of an ordinal scale with potentially unequal ‘jumps'.

Although PROMs are useful, they can be burdensome to both patients and clinicians. This is especially true for long questionnaires or when multiple PROMs are required to be administered. Response fatigue can be a serious problem for PROMs, as this can lead to missing data and bias, and reduce the willingness of patients to engage (Lavrakas, 2008). A Cochrane systematic review found that shorter questionnaires were associated with higher odds of completion (Edwards et al., 2009).

Computerized adaptive testing (CAT) is a form of artificial intelligence that can predict a patient's full-length PROM score based on an individualized subset of PROM questions. Adapting the CAT version of the questionnaire to an individual's real-time response may reduce the question burden while preserving accurate outcome data. Once IRT fit is achieved, a CAT can be developed.

The Patient Evaluation Measure (PEM) is a widely used hand-specific PROM for measuring the impact of surgery on hand function (Dias et al., 2001; Macey et al., 1995). Previously, it has been shown that CAT delivery of the PEM can be achieved for patients with thumb base osteoarthritis (Kamran et al., 2022). This study aimed to develop and evaluate the performance of a CAT version of the PEM for patients with CuTS.

## Methods

### Study design and setting

This study used data from the United Kingdom Hand Registry (UKHR) database, a voluntary national registry to evaluate outcomes for hand and wrist interventions. Patients who agreed to enter the registry were asked to complete the PEM at baseline and at predefined time points (3, 6 and 12 months) after surgery. By default, data in the registry were collected by email. For patients without an email, PEM responses could be captured using mail or Short Message Service (SMS). Results are collated by a central administrator independent of the operating surgeons.

Each patient provided written consent before inclusion into the registry, where the primary purpose is quality assurance of UK hand surgery. Secondary research use of the anonymized data collected and controlled by a registered charity is exempt from ethical approval in the UK. This was confirmed by the University of Oxford Clinical Trials and Research Governance before the start of this study.

### Patients

All consecutive adult patients who entered the registry between February 2012 and April 2019 and were diagnosed with CuTS were evaluated for eligibility. Between 2012 and 2017, the UKHR captured the original 10-item version of the PEM (Macey et al., 1995). This was changed in 2017 to the updated 11-item version of the PEM instead (Dias et al., 2001). The 11-item is identical to the 10-item version, except for an additional question concerning the duration of pain. As this item was missing for most of the patients in the registry, we chose to use complete response sets of the original 10-item version for the analysis.

### IRT and CAT

A CAT algorithm was developed using R statistical software based on an IRT model calibrated to the available PEM response sets. The data were fitted to an IRT model that handles ordinal response options, as presented here (the graded response model (GRM)). This was used to program a CAT algorithm. In-depth explanations of the assumption testing, fit statistics and model parameters for the modelling are provided in the Supplementary appendix S1, along with their results.

Next, a simulated dataset of 1000 PEM response sets was created based on the distribution of scores in the original dataset. The CAT algorithm was applied to a simulated dataset in a Monte Carlo simulation (Harrison et al., 2021). This allowed examination of the CAT performance when used in a distinct new population of individuals who behaved comparably to UK CuTS patients. For each simulated respondent, the CAT analysed individual responses one at a time, as if it were administering the questions in a real-life setting. After each response, the CAT predicted the respondent's total score and selected the next most informative question to administer. The CAT continued administering questions to the simulated patients with increasing precision (decreasing standard error of measurement (SEm)) until a prespecified precision threshold was met (SEm < 0.3). This precision threshold is similar to the measurement precision obtained in the Patient-Reported Outcome Measurement Information System (PROMIS) instruments (Gibbons et al., 2011; Reeve et al., 2007).

The θ scores (classically measured in logits) were rescaled so that they ranged from 0 to 100 for ease of interpretation in clinical practice and research.

### Measuring CAT performance

For each simulated respondent, the number of items needed to reach a precision of SEm < 0.3 was recorded, and the CAT score was compared with the full-length IRT questionnaire score. The following techniques were used to determine how closely the CAT-based scores reproduced the full-length IRT questionnaire scores.

1. The distribution of the CAT and full-length IRT questionnaire scores were plotted, and the mean error, absolute mean error and root square mean error were calculated.
2. The Pearson's correlation coefficient, intraclass correlation coefficient (ICC) and explained variance (R^2^) from the linear regression model (in which the CAT scores were regressed on the full-length IRT score) were calculated.
3. Bland–Altman analysis was performed, including the calculation of 95% limits of agreement, which describes the margin within which 95% of CAT score and full-length IRT questionnaire score aligned (Bland and Altman, 1986).

All analyses were performed in R statistical software (v 4.0.1) (R Foundation for Statistical Programming, Vienna).

## Results

### Participants

A total of 979 complete patient responses (from 522 distinct patients) were used for the analyses. In addition to PEM responses, sex, age and the type of treatment were available for most patients. Patient characteristics are shown in Table 1.

**Table 1.** Characteristics of 522 patients (979 complete responses) with cubital tunnel syndrome.

Characteristic	Value^a^
Age at operation, median (interquartile range)	53 (42–63)
Sex	
Female	222
Male	284
Unknown	16
Operation	
Simple decompression	414
Decompression with subcutaneous transposition	80
Decompression with submuscular transposition	5
Medial epicondylectomy	9
Revision surgery	14

^a^The *N* is displayed unless stated otherwise.

### Item reduction

**Figure 1.**
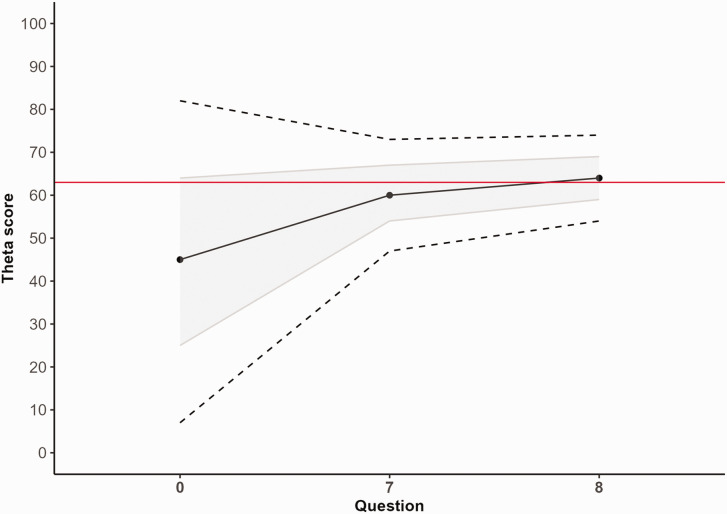
Example of the change in theta score (range 0–100) estimation in a patient during the computerized adaptive test (CAT). The red line represents the full-length score (63) if this patient would have filled in all 10 questions. Before the patient has answered any questions, the score starts at the population's average of 45. After completing the first question (PEM question 7), the score adjusts to 60 with a decreasing SEm. After the second question (PEM question 8), the score is 64 after which the CAT stops as the SEm has become smaller than 0.3. The grey area reflects the mean SD SEm, and the dashed lines represent SD 0.96 × SEm (95% confidence interval).

The CAT reduced the full-length PEM from ten items to a median of two questions (IQR 2–3; absolute range 2–4 questions) (Figure 1). This is an average reduction of 80%. In each case, the first item posed was ‘*For everyday activities, my hand is now*’ [answer options: No problem – Useless]. This is because the IRT model identified this as the most informative item in this population. The second most informative item was ‘*For my work, my hand is now*’ [answer options: No problem – Useless], which was administered to 932/1000 patients (93%). The administration frequency per question is shown in Table 2.

**Table 2.** Frequency table of the questions that were administered during the computerized adaptive testing (CAT) model in a simulated cohort of 1000 patients.

Description, [answer options range]	Administered in CAT, *N* (%)
Q1: Feeling in the hand is now [Normal – Absent]	87 (8.7%)
Q2: Pain when the hand is cold/damp [Non-existent – Unbearable]	12 (1.2%)
Q3: Pain in the hand most of the time [Non-existent – Unbearable]	28 (2.8%)
Q4: Ability to use the hand for fiddly things [Skilful – Clumsy]	256 (26%)
Q5: General movement of the hand [Flexible – Stiff]	33 (3.3%)
Q6: Hand grip [Strong – Weak]	132 (13%)
Q7: Hand usage for everyday activities [No problem – Useless]	1000 (100%)
Q8: Hand usage for work [No problem – Useless]	932 (93%)
Q9: Feeling when looking at hand appearance [Unconcerned – Embarrassed and self-conscious]	0 (0%)
Q10: Feeling when thinking about the hand [Unconcerned – Very upset]	0 (0%)

### Agreement between scores

**Figure 2.**
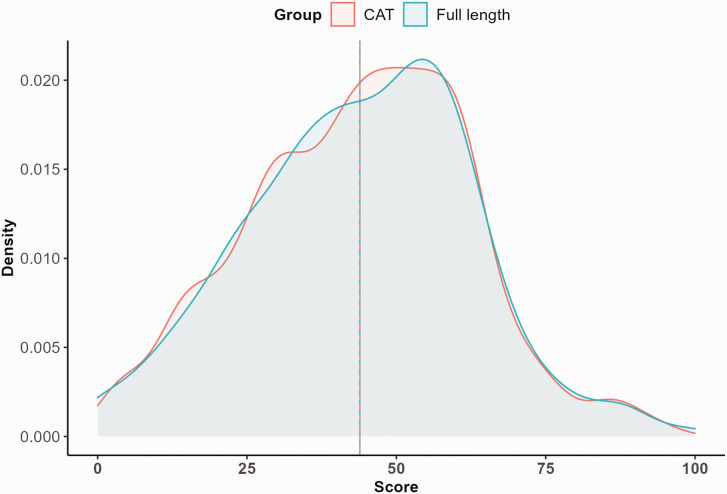
Distribution of the theta scores for the computerized adaptive testing (CAT) and full-length questionnaire (range 0–100). The vertical lines represent the mean values (43.9 versus 43.9, respectively) for both groups.

The distributions of the CAT scores and full-length IRT questionnaire scores are presented in Figure 2. The scores in both groups were similar (mean 43.9 (SD 18.4) versus mean 43.9 (SD 18.7), respectively), with a mean error of 0.08. The mean absolute error (which treats all differences as positive) was 2.90 and the root mean square error (which penalizes individual high errors to a greater extent) was 3.64.

**Figure 3.**
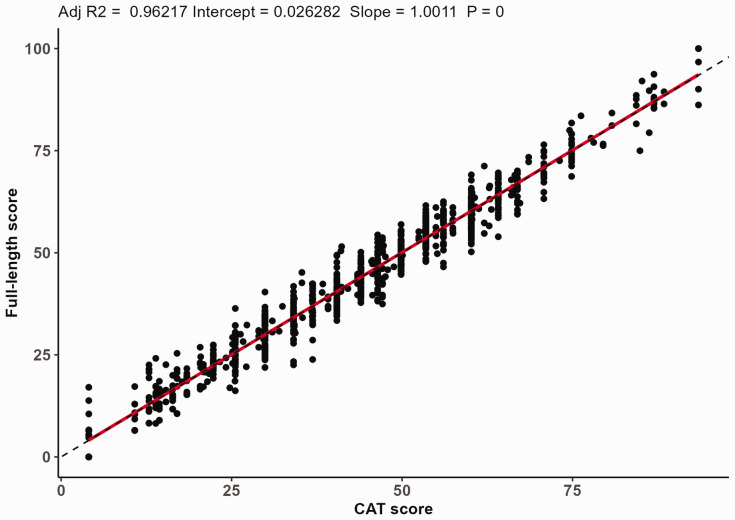
Linear regression of the computerized adaptive testing (CAT) scores versus the full-length scores, with an intercept of 0.03, a beta-coefficient of 1.00 and an explained variance (R^2^) of 0.96.

There was a strong linear relationship between the CAT scores and full-length IRT questionnaire scores, as indicated by a Pearson's correlation coefficient of 0.98, an ICC of 1.00 and an R^2^ of 0.96 from the linear regression model (Figure 3).

**Figure 4.**
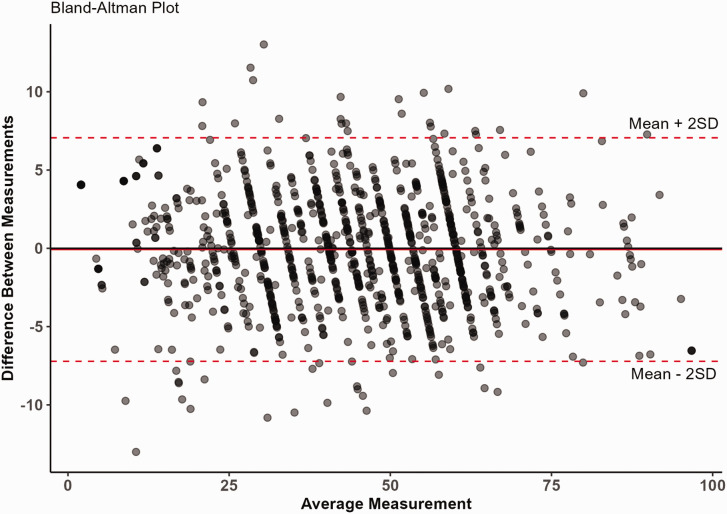
Bland–Altman plot. Differences between the computerized adaptive testing (CAT) and the full-length theta scores (range 0–100). The solid red line resembles a mean difference between the two scores of –0.07. The dashed red lines resemble the 95% limits of agreement, ranging from –7.21 to +7.06. The points remain in the same general pattern for all values on the *X*-axis, indicating that the agreement is constant for low and high scorers.

The Bland–Altman analysis (Figure 4) demonstrated little difference in agreement between low and high scorers. For 95% of the cases, a simulated respondent's CAT score was between −7.21 and −7.06 of the full-length IRT questionnaire score.

## Discussion

In this study in patients undergoing CuTS surgery, we were able to reduce the length of the PEM questionnaire by 80% (from ten questions to two) while enhancing its construct validity using modern psychometrics. First, we fitted PEM responses in CuTS patients to an IRT model. In contrast to the ordinal PEM scores, the IRT-based scoring quantifies individual measurement error, is on a continuous scale and accounts for the unequal weightings of the items. We have made these model parameters freely available, which can be used together with free R packages to rescore PEM response sets. Second, we developed a CAT version of the PEM based on the IRT model that considerably reduces the questionnaire's length while maintaining the enhanced validity of IRT scoring (demonstrated by the close relationship between CAT scores and full-length IRT questionnaire scores). The CAT can be a valid alternative to the full-length PEM in future research on CuTS and clinical practice. We plan to deploy this back into the UKHR, where the data to develop this was sourced.

One challenge for voluntary registries like UKHR is that they rely on patient engagement to maximize data capture. There rarely are resources available to support this with clinical staffing. It is hoped that making outcome measurement quick, easy and low in burden will facilitate better data capture with less attrition. This would have benefits in both research and clinical practice.

We have previously successfully developed a CAT version of the PEM for patients with trapeziometacarpal osteoarthritis (Kamran et al., 2022). The two CATs had a similar performance in item reduction, ranking the most informative items and agreement to full-length scores. However, the precise model parameters differ for these different conditions. Further investigation of different hand conditions would allow a better understanding of similarities and differences in measurement validity terms. While accurate measurement is desirable, it is also useful to establish whether parsimonious measurement solutions are possible, rather than needing many different models for different situations.

We have provided all the necessary data to operationalize the CAT as a smartphone application. This application could facilitate frequent (day-to-day) PEM sampling to monitor patients after treatment and to compare treatment regimens in clinical trials. Frequent PEM sampling would provide much richer data as it captures day-to-day variations while outcomes are less influenced by a single outlier. This might be important as symptoms of hand conditions often are dynamic (Harrison et al., 2020).

This study has limitations. First, due to the retrospective study design, we were unable to prove that the item reduction in the CAT also resulted in a higher completion rate and shorter completion time compared with the full-length questionnaire. Future prospective research should investigate this. Second, the CAT was developed in patients from the UKHR, but the software has not yet been validated in patients from other countries. Third, the CAT version of the PEM still relies on the content of the original PEM and suffers from the same potential limitations of content validity. The PEM was designed and deemed valid for usage across numerous hand and wrist conditions but not specifically for CuTS. Therefore, it may be incapable of detecting subtle changes in symptoms in some patients. Also, the PEM is not often used in ulnar nerve studies, studies using this CAT will be less comparable with previous research in CuTS. However, PEM remains the core outcome measure of the UKHR, which was the data source here. Its ongoing use and popularity provide an indication that it is considered relevant for use in CuTS in terms of face validity. Other PROMs that are more frequently used in CuTS include the Boston Carpal Tunnel Questionnaire (BCTQ), which is International Consortium for Health Outcomes Measurements (ICHOM's) recommended PROM for nerve conditions, and the patient-rated ulnar nerve evaluation (PRUNE) (Levine et al., 1993; MacDermid and Grewal, 2013). These questionnaires may be more sensitive to change than the CAT version of the PEM, however, they also come at a higher patient burden of 19 and 20 questions, respectively, and do not benefit from modern IRT. Developing CAT versions for these questionnaires will be even more conceptually and technically demanding than in the current study, as the BCTQ and PRUNE do not meet the unidimensionality criteria (Lue et al., 2015; MacDermid and Grewal, 2013). As such, more advanced models are needed to account for multidimensionality, as explained in a previous article (Harrison et al., 2022). Last, PEM is widely used in other hand conditions, supporting comparisons of hand functions impairment and outcome across hand conditions, which would not be possible with, for example, BCTQ.

Future research will focus on the feasibility of administering CAT versions on the PEM using smartphone applications for clinical trials and registries, and on external validation for other populations.

## Supplemental Material

sj-pdf-1-jhs-10.1177_17531934231164959 - Supplemental material for Computerized adaptive testing for the patient evaluation measure (PEM) in patients undergoing cubital tunnel syndrome surgery Click here for additional data file. Supplemental material, sj-pdf-1-jhs-10.1177_17531934231164959 for Computerized adaptive testing for the patient evaluation measure (PEM) in patients undergoing cubital tunnel syndrome surgery by Joris S. Teunissen, Steven E. R. Hovius, Dietmar J. O. Ulrich, Fadi Issa, Jeremy N. Rodrigues, Conrad J. Harrison in Journal of Hand Surgery (European Volume)
