# Supplementary material for: Computerized adaptive testing for the patient evaluation measure (PEM) in patients undergoing cubital tunnel syndrome surgery
Source: J Hand Surg Eur Vol. 2023 Apr 17;48(10):1042–7. doi: 10.1177/17531934231164959 (PMC10616996; doi:10.1177/17531934231164959)
Supplement: sj-pdf-1-jhs-10.1177_17531934231164959 - Supplemental material for Computerized adaptive testing for the patient evaluation measure (PEM) in patients undergoing cubital tunnel syndrome surgery [file sj-pdf-1-jhs-10.1177_17531934231164959.pdf]

SUPPLEMENTARY FILE

Computerized adaptive testing shortens the PEM PROM in cubital tunnel syndrome by 80% while improving validity

SUPPLEMENTARY METHODS

Item response theory (IRT) - model assumptions

Before constructing a CAT, the items of the PEM were evaluated for suitability for the construction of an IRT model. Therefore, the four key assumptions of the IRT framework were checked: 1) unidimensionality; 2) local independence; 3) monotonicity; and 4) item invariance.

1. Unidimensionality

Unidimensionality means that items measure a single trait, in this case, hand function. Unidimensionality was evaluated through Mokken analysis (Mokken, 2011). Unidimensionality was confirmed when the automatic item selection procedure (AISP) grouped all the items into one monotonic scale.

2. Local independence

Local independence means that items within a scale are unrelated except for their measurement of the single underlying trait. Local independence was evaluated by analysing Yen's Q3 residual covariance matrix using a cutoff value of 0.2 to demonstrate local dependency (Christensen et al., 2017; Gaskin and Happell, 2014).

3. Monotonicity

Monotonicity means that the probability of endorsing an item continuously increases as the trait level increases. Monotonicity was evaluated using Mokken analysis on the item level and for the

overall model. The assumption was considered satisfied when the individual items had a scalability coefficient (H_i) > 0.30 and the overall model (H) > 0.50 (Mokken, 2011).

4. Measurement invariance

Measurement invariance means that estimated item parameters are constant across different subgroups of patients. Differential item functioning by age (median split: <53 years versus ≥53 years) and gender was evaluated with the R package "*Lordif*" (version 0.3–3) (Choi et al., 2011). The assumption was considered satisfied when the models' change in R^2 was ≤ 0.02.

Item-level and Scale-level Fit Statistics

After evaluation of the assumptions for IRT, a graded response model (GRM) was fit for the item response data using the "*mirt"* package in R.

A generalization of Orlando and Thissen's S-X^2 for polytomous data was used to identify how well items fitted the GRM on item level (Orlando and Thissen, 2003). Items with an S-X^2 p-value <0.001 were considered poor fitting. The following parameters were considered to evaluate scale level fit statistics: 1) comparative fit index (CFI; ≥0.95); 2) Tucker–Lewis index (TLI; ≥0.95); 3) root mean square error of approximation (RMSEA; <0.06); 4) and standardized root mean squared residual statistics (SRMSR; <0.08). Furthermore, infit and outfits statistics (item level fit) were computed (Linacre, 2002).

SUPPLEMENTARY RESULTS

1. Unidimensionality

All items were grouped into one monotonic scale, suggesting that the assumption of unidimensionality was met.

2. Local independence

The residual correlations ranged from -0.31 to 0.62. Of the 45 residual correlations, two (4.4%) (PEM item 3 with PEM item 4, and PEM item 9 with PEM item 10) had a value larger than 0.2, suggesting local dependence. However, this was not severe enough to remove the item from the model.

3. Monotonicity

For the individual items, the scalability coefficients (H_i) ranged from 0.478 (PEM item 9) to 0.727 (PEM item 7) (Table S2). The overall scalability coefficient (H) was 0.631. Therefore, the monotonicity assumption was met.

4. Measurement invariance

Sex was known for 506/522 (96%) of the respondents. Age was known for 502/522 (96%) of the respondents. In both models the R^2 was 0.0001, meaning that the assumption was met.

Item-level and Scale-level fit statistics

The parameters of the GRM are shown in Table S1. The infit and outfit statistics are shown in Table S2. The model had reasonable overall fit with a CFI of 0.953, TLI of 0.939, RMSEA of 0.151, and SRMSR of 0.064

SUPPLEMENTARY REFERENCES

Choi SW, Gibbons LE, Crane PK. Lordif: An R package for detecting differential item functioning using iterative hybrid ordinal logistic regression/item response theory and Monte Carlo simulations. J Stat Softw. 2011, 39: 1.

Christensen KB, Makransky G, Horton M. Critical Values for Yen's Q(3): Identification of Local Dependence in the Rasch Model Using Residual Correlations. Appl Psychol Meas. 2017, 41: 178–94.

Gaskin CJ, Happell B. On exploratory factor analysis: a review of recent evidence, an assessment of current practice, and recommendations for future use. Int J Nurs Stud. 2014, 51: 511–21.

Linacre JM. What do infit and outfit, mean-square and standardized mean. Rasch Meas Trans. 2002, 16: 878.

Mokken RJ. A theory and procedure of scale analysis. *A theory Proced. scale Anal.* De Gruyter Mouton, 2011.

Orlando M, Thissen D. Further investigation of the performance of S-X2: An item fit index for use with dichotomous item response theory models. Appl Psychol Meas. 2003, 27: 289–98.

SUPPLEMENTARY TABLES

Table S1. Results concerning monotonicity, GRM fit at the item level, and GRM parameters.

Item	Monotonicity	GRM model fit	GRM item parameters						
**ID**	**Loevinger's H_i**	**S-X^2 p-value**	**a**	**b1**	**b2**	**b3**	**b4**	**b5**	**b6**
**PEM 1**	0.620	0.005	2.276	-1.712	-1.013	-0.335	0.275	0.820	1.296
**PEM 2**	0.567	0.000	1.732	-1.365	-0.564	0.065	0.663	1.559	2.355
**PEM 3**	0.615	0.025	2.069	-1.153	-0.337	0.282	0.923	1.704	2.523
**PEM 4**	0.684	0.109	3.250	-1.484	-0.760	-0.246	0.115	0.573	1.002
**PEM 5**	0.645	0.004	2.784	-0.821	-0.289	0.175	0.668	1.291	1.730
**PEM 6**	0.648	0.330	2.907	-1.445	-0.696	-0.137	0.324	0.825	1.262
**PEM 7**	0.727	0.267	5.798	-1.104	-0.445	0.070	0.592	1.230	1.786
**PEM 8**	0.690	0.045	3.993	-1.209	-0.573	-0.026	0.487	1.125	1.626
**PEM 9**	0.478	0.031	1.353	0.076	0.746	1.133	1.688	2.407	2.984
**PEM 10**	0.613	0.275	2.201	-0.963	-0.375	0.219	0.762	1.428	1.935

Abbreviation: PEM = Patient Evaluation Measure.

Table S2. Infit and outfit statistics for the PEM items.

Item	Outfit	Infit
PEM 1	1.070	1.043
PEM 2	0.968	0.994
PEM 3	0.922	0.975
PEM 5	0.788	0.885
PEM 6	0.902	0.979
PEM 7	0.929	0.938
PEM 8	0.659	0.717
PEM 9	1.009	0.989
PEM 10	0.840	0.957
PEM 11	0.955	1.023

Abbreviation: PEM = Patient Evaluation Measure.
